# Gut Bacteria Associated With *Monochamus saltuarius* (Coleoptera: Cerambycidae) and Their Possible Roles in Host Plant Adaptations

**DOI:** 10.3389/fmicb.2021.687211

**Published:** 2021-06-21

**Authors:** Si-Xun Ge, Feng-Ming Shi, Jia-He Pei, Ze-Hai Hou, Shi-Xiang Zong, Li-Li Ren

**Affiliations:** ^1^Beijing Key Laboratory for Forest Pest Control, Beijing Forestry University, Beijing, China; ^2^Sino-French Joint Laboratory for Invasive Forest Pests in Eurasia, French National Research Institute for Agriculture, Food and Environment (INRAE), Beijing Forestry University, Beijing, China

**Keywords:** microbiota, metabolomics, host adaptation, host-microbe interaction, intestinal bacterial composition, borer

## Abstract

*Monochamus saltuarius* (Coleoptera: Cerambycidae) is an important native pest in the pine forests of northeast China and a dispersing vector of an invasive species *Bursaphelenchus xylophilus*. To investigate the bacterial gut diversity of *M. saltuarius* larvae in different host species, and infer the role of symbiotic bacteria in host adaptation, we used 16S rRNA gene Illumina sequencing and liquid chromatography-mass spectrometry metabolomics processing to obtain and compare the composition of the bacterial community and metabolites in the midguts of larvae feeding on three host tree species: *Pinus koraiensis*, *Pinus sylvestris* var. *mongolica*, and *Pinus tabuliformis.* Metabolomics in xylem samples from the three aforementioned hosts were also performed. Proteobacteria and Firmicutes were the predominant bacterial phyla in the larval gut. At the genus level, *Klebsiella*, unclassified_f__*Enterobacteriaceae*, *Lactococcus*, and *Burkholderia*–*Caballeronia*–*Paraburkholderia* were most dominant in *P. koraiensis* and *P. sylvestris* var. *mongolica* feeders, while *Burkholderia*–*Caballeronia*–*Paraburkholderia*, *Dyella*, *Pseudoxanthomonas*, and *Mycobacterium* were most dominant in *P. tabuliformis* feeders. Bacterial communities were similar in diversity in *P. koraiensis* and *P. sylvestris* var. *mongolica* feeders, while communities were highly diverse in *P. tabuliformis* feeders. Compared with the other two tree species, *P. tabuliformis* xylems had more diverse and abundant secondary metabolites, while larvae feeding on these trees had a stronger metabolic capacity for secondary metabolites than the other two host feeders. Correlation analysis of the association of microorganisms with metabolic features showed that dominant bacterial genera in *P. tabuliformis* feeders were more negatively correlated with plant secondary metabolites than those of other host tree feeders.

## Introduction

Xylophagous beetles are insects that feed mainly on woody tissues beneath the bark of trees, including phloem and xylem. These wood-feeding insects encounter a particularly challenging environment because of extremely low quantities of nitrogen (Hölttä et al., 2013), poorly accessible carbohydrate sources in the form of lignocellulose (Scully et al., 2013; Tokuda et al., 2014), and a considerable amount of secondary defense metabolites (Ericsson et al., 1988; Keeling and Bohlmann, 2006). However, xylophagous beetles do not face these challenges alone but are supported by their gut microbes. Wood-feeding beetles harbor a highly diverse gut microbiome community (Reid et al., 2011). The symbiotic microorganisms represented by intestinal bacteria have repeatedly been shown to facilitate interactions between trees and wood feeders by supplementing essential nutrients or degrading complex dietary polymers (Geib et al., 2008; Boone et al., 2013). Recently, known microbial functions were expanded to include degradation of phytochemicals and even phytochemical utilization to increase the nutritional properties of the beetle diet (Berasategui et al., 2017).

The diversity of gut bacteria in phytophagous insects is largely affected by the host plant. Different host plants result in different nutritional compositions and intestinal environments, which can significantly influence the insect’s bacterial gut symbionts (Kim et al., 2017; Scully et al., 2018; Santos-Garcia et al., 2020). For example, bacterial symbionts associated with the guts of the western corn rootworm (Dematheis et al., 2012; Chu et al., 2013), the Colorado potato beetle (Chung et al., 2017; Wang et al., 2020), and *Cephaloleia* spp. (Blankenchip et al., 2018) can shift in response to different dietary stimuli. Bacteria inhabiting the gut lumen of insects play an important role in insect–host plant adaptation. On the one hand, this is mainly because they colonize the organ where food is assimilated, and therefore can respond relatively quickly to environmental changes (e.g., changes in host plants) (Santos-Garcia et al., 2020); on the other, these symbionts can potentially contribute to host plant specificity by providing nutrients lacking in specific plant species, thereby allowing an insect to develop and reproduce on a plant that is nutritionally unsuitable (Hansen and Moran, 2014). Also, gut-associated bacteria have a dynamic microbiome because they contact free-living external bacteria and can acquire novel capabilities, such as genes encoding detoxifying enzymes, or can even be replaced by new bacteria with different metabolic potential that could enhance host fitness (Hansen and Moran, 2014).

Although plants represent the largest source of biomass in terrestrial systems, their tissues are largely composed of recalcitrant molecules and many chemical defense substances that can direct toxicity, induce oxidative stress, damage epithelial cells, and disrupt digestion (Watanabe and Tokuda, 2010; Mason, 2020). While the role of insect gut microbes in degrading secondary plant metabolites and resisting plant defenses has been repeatedly reported, the presence of microbes seems to augment the intrinsic capacity of insects to contend with plant defenses. For example, leguminous plants can produce protease inhibitors, which prevent protein digestion. However, bacterial species isolated from the *Anticarsia gemmatalis* (moth that feeds on leguminous plants) gut secrete proteases that are relatively unaffected by the protease inhibitors present in plant tissues (Pilon et al., 2013). In coniferous tree species, various chemical defense substances play an important role in the process of pest prevention (Martin et al., 2004). While metabolism of chemicals by the insect gut microbiome is frequently observed in conifer-attacking insects (Boone et al., 2013; Xu et al., 2015; Berasategui et al., 2017), the gut bacteria of xylophagous beetles can effectively help beetles resist chemical plant defenses: gut bacteria of the pine weevil (*Hylobius abietis*) can help weevils metabolize a variety of conifer diterpenes, and gut bacteria can even utilize diterpenoids as a carbon source, and may produce nutrients to increase insect fitness (Berasategui et al., 2017). Observations of such examples fall short of proving that gut bacteria are crucial to overcome plant defenses, but they do provide suggestive evidence for such a role (Hansen and Moran, 2014).

*Monochamus saltuarius* (Coleoptera: Cerambycidae) has a wide range of host plants in conifer species. It is regarded as a severe forest pest in South Korea, Japan, and northeast China because it is a vector for an invasive pathogenic nematode, *Bursaphelenchus xylophilus*, which causes pine wilt disease (Takizawa and Shoji, 1982; Kwon et al., 2006; Yu and Wu, 2018; Hou et al., 2021). As the insect vector of *B. xylophilus* in northeast China, the wide adaptation of *M. saltuarius* to different host plants greatly facilitates interspecies transmission of *B. xylophilus.* Therefore, studying the adaptations of *M. saltuarius* to different host plants is of great significance for guiding the prevention and control of pine wilt disease. Although *M. saltuarius* can complete its life cycle in all seven major host tree species in China (*Larix kaempferi*, *Pinus armandii*, *Pinus bungeana*, *Pinus koraiensis*, *Pinus massoniana*, *Pinus sylvestris* var. *mongolica*, and *Pinus tabuliformis*), it has obvious feeding and oviposition preference for different host trees. Compared with the other five host tree species, *M. saltuarius* has a significant preference for laying eggs on *P. koraiensis* and *P. massoniana* (unpublished data); the feeding preference of adults for three host tree species from high to low is *P. koraiensis*, *P. tabuliformis*, and *L. kaempferi* (Pan et al., 2020). In addition, larvae of *M. saltuarius* feeding on different host tree species also show significant differences in body size or even in survival rates (Hwang et al., 2008).

Although some studies on the gut bacteria of *Monochamus* species have been carried out, there are still many issues to be resolved. At present, there are no reports on bacteria associated with *M. saltuarius*; whether the microbial composition of larvae feeding on different host tree species is diverse; the potential function of intestinal bacteria; or the role of intestinal bacteria in adapting to different host plants. This research was devoted to investigating the intestinal bacteria of *M. saltuarius* with regard to the above aspects. In this study, *M. saltuarius* larvae feeding on three different host tree species (*P. koraiensis*, *P. sylvestris* var. *mongolica*, and *P. tabuliformis*) and tissues of these host trees were systematically sampled during the same time period at the same location. Bacterial compositions of individual beetles feeding on different host trees were determined by sequencing to explore the taxonomic diversity in different groups. The composition of each compound in the *M. saltuarius* midgut and tissues of its host trees were analyzed by liquid chromatography (LC)-mass spectrometry (MS) methods. The bacterial communities associated with *M. saltuarius* were characterized. Correlation analysis of associations between microorganisms and metabolite features provides a comprehensive understanding of the composition and function of microbial communities, and can help us further understand the interaction between the gut microbes of larvae and the host tree species they feed on; in this manner, a combination analysis based on a metagenomic approach and metabolomic methods was conducted to reveal the possible role of intestinal microorganisms in strategies for adapting to different host tree species.

## Materials and Methods

### Sample Collection and Dissections

All samples were collected from mixed pine forests at Cangshi Forest Farm, Hongtoushan Town, Qingyuan Manchu Autonomous County, Fushun City, Liaoning Province, China, in August and September 2020. Trees with yellow-green needles and oviposition scars from *M. saltuarius* were selected to collect larvae and wood tissues. Longitude and latitude data for different tree species were collected as follows: *P. koraiensis* (124.5218°E 41.9829°N), *P. sylvestris* var. *mongolica* (124.5202°E 41.9803°N), and *P. tabuliformis* (124.4995°E 41.9822°N). Healthy *M. saltuarius* larvae of instar III or IV were collected from the xylem galleries of selected host trees using sterile fine-tipped forceps and then transferred into a sterile 5 mL centrifuge tube. Individual larvae from different host species were collected and numbered from 1 to 9; thus larvae from *P. koraiensis* were designated PK1–PK9, and larvae from *P. tabuliformis* were designated PT1–PT8. We divided all collected larvae into two groups for 16S rRNA gene Illumina sequencing and LC–MS metabolomics processing, such that the same sample names appeared in the microbiome and metabolomics analyses, but they represented different individuals from the same host tree species. Since only 11 larvae were collected from the xylem of *P. sylvestris* var. *mongolica*, only five individuals were used for 16S rRNA gene Illumina sequencing and six for metabolomics analysis (PK5, PT4, and PT5 were excluded from 16S rRNA gene Illumina sequencing because of slight contamination). Wood tissues were collected from the xylem of each tree species around larval galleries using sterile fine-tipped forceps and were transferred into sterile 5 mL centrifuge tubes; 10 grams of wood tissue samples were collected from different tree species, and a total of eight replicates of each tree species were carried out, each replicate was accurately weighed as 50 mg; xylem samples from *P. koraiensis* were designated as T_PK1–T_PK8, and *P. tabuliformis* samples were designated as T_PT1–T_PT8, while *P. sylvestris* var. *mongolica* samples were designated as T_PS1–T_PS8. Both larvae and wood tissue samples were placed on dry ice immediately after collection, brought back to the laboratory, and then stored at −80°C until use.

*M. saltuarius* larvae were surface sterilized with 70% ethanol for 1 min and rinsed twice with sterile water before dissection. Insects were dissected under aseptic conditions using sterilized dissection scissors and fine-tipped forceps to obtain the midgut. Midguts were transferred individually into 1.5-mL microfuge tubes containing 0.5 mL PBS. Guts were sonicated in the tubes (50/60 Hz, 117 V, 1.0 A; Branson Ultrasonics, Danbury, CT, United States) for 30 s, macerated with a plastic pestle, and vortexed at medium speed for 10 s to separate bacterial cells from the gut wall (Schloss et al., 2006). The tubes were then centrifuged at low speed (1,000 rpm), and the supernatants were collected for bacterial DNA extraction.

### DNA Extraction, Quantitative PCR, 16S rRNA Gene Amplification, and Sequencing

Microbial community genomic DNA was extracted from *M. saltuarius* larval midgut samples using the DNeasy^®^ 96 PowerSoil^®^ Pro QIAcube^®^ HT kit (Qiagen, Hilden, Germany), according to the manufacturer’s instructions. DNA extractions were checked on a 1% agarose gel, and DNA concentration and purity were determined with a NanoDrop 2000 UV-Vis spectrophotometer (Thermo Fisher Scientific, Wilmington, NC, United States). The hypervariable region V3–V4 of the bacterial 16S rRNA gene was amplified with primer pairs 338F (5′-ACTCCTACGGGAGGCAGCAG-3′) and 806R (5′-GGACTACHVGGGTWTCTAAT-3′) using an ABI GeneAmp^®^ 9700 PCR thermocycler (ABI, CA, United States).

Absolute quantification of bacteria was carried out using qPCR targeting the 16S rRNA gene to estimate the abundance of bacteria in each sample. Per reaction, a mix of 10 μL ChamQ SYBR Color qPCR Master Mix (2X) (Vazyme Biotech Co., Ltd.), 0.25 μL 5 μM forward primer, 0.25 μL 5 μM reverse primer, 7.5 μL nuclease-free water and 2 μL DNA template was added. qPCR was performed in a LineGene 9600 Plus fluorescent quantitative PCR detection system (Hangzhou Bioer technology, Co., Ltd.), as follows: initial denaturation at 95°C for 5 min, then 40 cycles of denaturation at 95°C (30 s), annealing at 56°C (30 s), and elongation at 72°C (40 s), followed by a melt curve analysis from 60 to 95°C in 0.5°C increments for 5 s each. Each constructed plasmid diluted by 10-fold serial dilution (90 μL diluent + 10 μL plasmid). 4–6 points (which contain standard sample concentration between 10^–2^ and 10^–7^) have been selected through preliminary study to make a standard curve. We required that all qPCR standard curves have efficiency values between 90 and 110% and R^2^ values above 0.9.

PCR amplification of the 16S rRNA gene was performed as follows: initial denaturation at 95°C for 3 min, followed by 30 cycles of denaturing at 95°C for 30 s, annealing at 55°C for 30 s, and extension at 72°C for 45 s, and a single extension at 72°C for 10 min, ending at 4°C. The PCR mixtures contained 5 × *TransStart* FastPfu buffer (4 μL), 2.5 mM dNTPs (2 μL), forward primer (5 μM, 0.8 μL), reverse primer (5 μM, 0.8 μL), *TransStart* FastPfu DNA Polymerase (0.4 μL), template DNA (10 ng), and finally ddH_2_O up to 20 μL. PCR reactions were performed in triplicate. The PCR product was extracted from a 2% agarose gel and purified using the AxyPrep DNA Gel Extraction kit (Axygen Biosciences, Union City, CA, United States), according to the manufacturer’s instructions, and quantified using the Quantus^TM^ Fluorometer (Promega, United States). Subsequently, purified amplicons were pooled at equimolar concentrations and paired-end sequenced on the Illumina MiSeq PE300 platform or the NovaSeq PE250 platform (Illumina, San Diego, CA, United States), according to the standard protocols of Majorbio Bio-Pharm Technology Co., Ltd. (Shanghai, China). Negative controls were included in every set of amplifications. No amplification products were observed in gels, and thus no negative controls were sequenced.

Sequencing libraries were generated using the NEXTFLEX^§^ Rapid DNA-Seq Kit for Illumina (Bioo Scientific, Austin, TX, United States) following the manufacturer’s recommendations and index codes were added. The library quality was assessed by the Qubit@ 2.0 Fluorometer (Thermo Scientific) and Agilent Bioanalyzer 2100 system.

### Sequence Processing and Analysis

Raw 16S rRNA gene sequencing reads were demultiplexed, quality filtered by fastp version 0.20.0 (Chen et al., 2018), and merged with FLASH version 1.2.7 (Magoč and Salzberg, 2011) with the following criteria: (i) the 300 bp reads were truncated at any site, receiving an average quality score of < 20 over a 50 bp sliding window, and truncated reads shorter than 50 bp were discarded, and reads containing ambiguous nucleotides were also discarded; (ii) only overlapping sequences longer than 10 bp were assembled according to the overlapping sequence (the maximum mismatch ratio of overlapping regions was 0.2; reads that could not be assembled were discarded); and (iii) samples were distinguished according to the barcode and primers, and the sequence direction was adjusted, with exact barcode matching, and a two-nucleotide mismatch was allowed in primer matching.

Operational taxonomic units (OTUs) with a similarity cutoff of 97% (Stackebrandt and Goebel, 1994; Edgar, 2013) were clustered using UPARSE version 7.1 (Edgar, 2013), and chimeric sequences were identified and removed. The taxonomy of each OTU representative sequence was analyzed by RDP Classifier version 2.2 (Wang et al., 2007) against the 16S rRNA database SILVA version 138 using a confidence threshold of 0.7.

Comparisons of qPCR results between groups were performed using Tukey’s multiple comparisons test in GraphPad Prism 8 (GraphPad Software, San Diego, CA, United States). 16S rRNA gene Illumina sequencing results was analyzed using the free online platform Majorbio Cloud Platform^1^. α-diversity indexes were calculated using mothur (version 1.30.2) (Schloss et al., 2009). The rarefaction curve and bar graphs were generated using the “vegan” package in R (Oksanen et al., 2010). β-diversity was estimated using QIIME (version 1.9.1) (Caporaso et al., 2010) and visualized using principal coordinate analysis, and the results were plotted using R. The Kruskal-Wallis *H*-test was used to identify phyla and genera that showed significant differences in abundance within groups. Functional contributions of various taxa to different KEGG ortholog groups were computed with the “metagenome_contrib” command of PICRUSt2 (Douglas et al., 2019), and were visualized as bar plots and heat maps.

### LC–MS Metabolomics Processing

Wood tissue samples (50 mg) were accurately weighed, and metabolites were extracted using a 400 μL methanol:water (4:1, vol/vol) solution. The mixture was allowed to settle at −20°C and was treated with a high-throughput tissue crusher (Wonbio-96c; Shanghai Wanbo Biotechnology Co., Ltd.) at 50 Hz for 6 min, followed by vortexing for 30 s and ultrasonication at 40 kHz for 30 min at 5°C. Samples were placed at −20°C for 30 min to precipitate proteins. After centrifugation at 13,000g and 4°C for 15 min, the supernatants were carefully transferred to sample vials for LC–MS/MS analysis. Chromatographic separation of the metabolites was performed on a Thermo UHPLC system equipped with an ACQUITY BEH C18 column (100 × 2.1 mm; inner diameter, 1.7 μM; Waters, Milford, CT, United States). The mobile phases consisted of 0.1% formic acid in water (solvent A) and 0.1% formic acid in acetonitrile:isopropanol (1:1, vol/vol) (solvent B). The solvent gradient was altered according to the following steps: from 0 to 3 min, 95% A:5% B to 80% A:20% B; from 3 to 9 min, 80% A:20% B to 5% A:95% B; from 9 to 13 min, 5% A:95% B to 5% A:95% B; from 13 to 13.1 min, 5% A:95% B to 95% A:5% B; and from 13.1 to 16 min, 95% A:5% B to 95% A:5% B for equilibrating the systems. The sample injection volume was 2 μL, and the flow rate was set to 0.4 mL/min. The column temperature was maintained at 40°C. During the analysis period, all samples were stored at 4°C. Mass spectrometric data were collected using a Thermo UHPLC-Q Exactive Mass Spectrometer equipped with an electrospray ionization source operating in either positive or negative ion mode. The optimal conditions were set as follows: Aus gas heater temperature, 400°C; sheath gas flow rate, 40 psi; Aus gas flow rate, 30 psi; ion-spray voltage floating, −2,800 V in negative mode and 3,500 V in positive mode; normalized collision energy, 20–40–60 V rolling for MS/MS. Data acquisition was performed with data-dependent acquisition mode. Detection was carried out over a mass range of 70–1,050 *m*/*z*. As part of the system conditioning and quality control (QC) process, a pooled QC sample was prepared by mixing equal volumes of all samples. The QC samples were treated and tested in the same manner as the analytic samples. This facilitated representation of the whole sample set, which would be injected at regular intervals (every eight samples) to monitor the stability of the analysis.

### Metabolomic Data Analysis

After ultra-performance LC coupled with time-of-flight MS analyses, raw data were imported into Progenesis QI 2.3 software (Non-linear Dynamics, Waters) for peak detection and alignment. Metabolic features detected at a rate of at least 80% in any set of samples were retained. After filtering, minimum metabolite values were imputed for specific samples in which the metabolite levels fell below the lower limit of quantitation, and each metabolic feature was normalized by sum. The internal standard was used for data QC (reproducibility); metabolic features with relative QC standard deviation > 30% were discarded. Following normalization procedures and imputation, statistical analysis was performed on log-transformed data to identify significant differences in metabolite levels between comparable groups. Mass spectra of these metabolic features were identified by using the accurate mass, MS/MS fragment spectra, and isotope ratio differences to search reliable biochemical databases such as the Human Metabolome Database ^2^ and the METLIN database^3^. The mass tolerance between the measured *m*/*z* values and the exact mass of the components of interest was ± 10 ppm. For metabolites with MS/MS confirmation, only those with MS/MS fragment scores > 30 were considered to be confidently identified. Otherwise, metabolites had only tentative assignments.

A multivariate statistical analysis was performed using the ropls^4^ (version 1.6.2) R package from Bioconductor on the Majorbio Cloud Platform^5^. Metabolites at differential levels between three groups in two types of samples were summarized individually and mapped into the pathway biosynthesis of other secondary metabolites, along with the metabolism of terpenoids and polyketides. The analysis was based on a database search (KEGG^6^). Heat maps of the metabolites in xylem samples annotated in the above pathways were generated with GraphPad Prism 8 (GraphPad Software, San Diego, CA, United States).

### Correlation Analysis Between Different Metabolites and Bacterial Communities

Differences in the metabolic profile of a microbial community reflect differences in the microbial community; thus we attempted to define relationships between microbial community structure and metabolic function based on microbial and metabolomic data. Correlations between different metabolites and bacterial communities were assessed by Spearman’s correlation analysis using the pheatmap package in R (Kolde, 2012); correlation analysis between the top five abundant bacterial genera in each group of host tree species feeders (Figure 8A) or highly abundant bacterial genera in intestinal samples of larvae feeding on *P. tabuliformis* (abundance ≥ 2%, except for the top five) (Figure 8B), and compounds annotated as related to plant defense substances (including metabolites involved in the following pathways: xenobiotic biodegradation and metabolism, metabolism of terpenoids and polyketides, and biosynthesis of other secondary metabolites) (Figure 8), are illustrated in heat maps. *P*-values were false discovery rate adjusted, and corrected *P* < 0.05, 0.01, and 0.001 were regarded as statistically significant, extremely significant, and most significant.

## Results

### General Profile of the OTU and qPCR Sequencing Data

**FIGURE 1:**
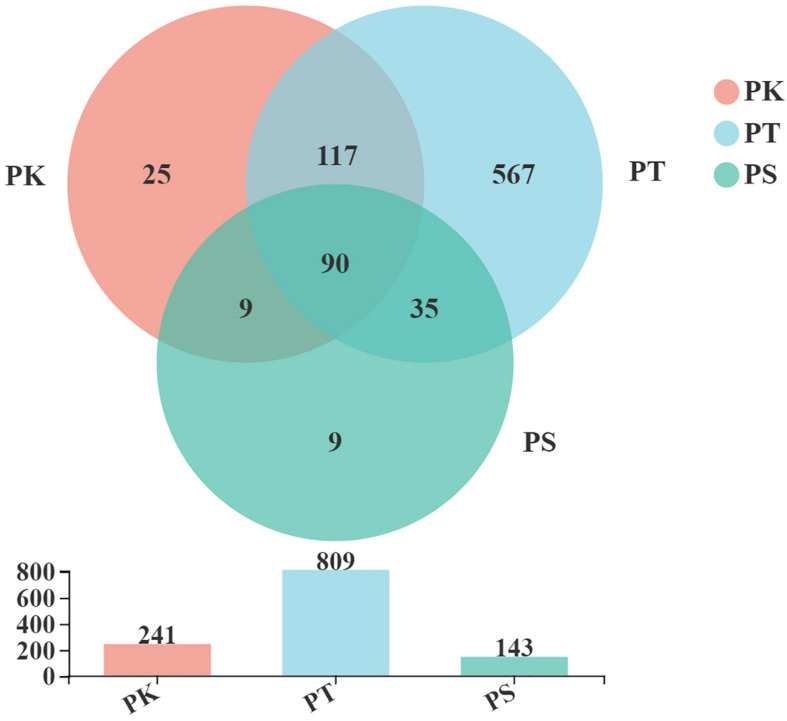
Venn diagrams of OTUs shared between intestines of larvae feeding on the three host tree species. PK, midguts of larvae feeding on *P. koraiensis*; PT, midguts of larvae feeding on *P. tabuliformis*; PS, midguts of larvae feeding on *P. sylvestris* var. *mongolica*. All of these abbreviations apply to the following figures.

A total of 925,336 high-quality and filtered sequences (an average of 48,702 reads per sample) from which 852 OTUs were obtained from 19 *M. saltuarius* larval midgut samples, with a median of 136 OTUs per sample (Supplementary Table 1). According to the rarefaction curves, the number of sequences obtained reflected the main bacterial information in each sample (Rarefaction curves: Supplementary Figure 1). There were 90 OTUs shared between all samples, and 161 OTUs were shared between two groups. Twenty-five and nine unique OTUs were detected in samples from larvae feeding on *P. koraiensis* and *P. sylvestris* var. *mongolica*, respectively. In total, 567 unique OTUs were found in larvae feeding on *P. tabuliformis* (Figure 1). Thus, the midguts of larvae feeding on *P. koraiensis* and *P. sylvestris* var. *mongolica* were similar in microbial composition, and the number of microbial OTUs in larvae feeding on *P. tabuliformis* was significantly greater than those in the two groups above, more than trebling that of larvae feeding on *P. koraiensis*.

Based on the qPCR results (Supplementary Figure 2 and Supplementary Table 2), the *P. koraiensis* feeding samples exhibited an average of 4.62122e + 09 copies/g of bacterial 16S rRNA genes per sample. The *P. tabuliformis* feeders exhibited an average of 3.29706e + 08 copies/g of bacterial 16S rRNA genes per sample and *P. sylvestris* var. *Mongolica* feeding samples exhibited an average of 3.74193e + 09 copies/g of bacterial 16S rRNA genes per sample. According to the results of Tukey’s multiple comparisons test between groups, no significant differences were observed (Supplementary Table 3).

### Comparisons of α- and β-Diversity in Samples From Different Host Tree Species

**FIGURE 2:**
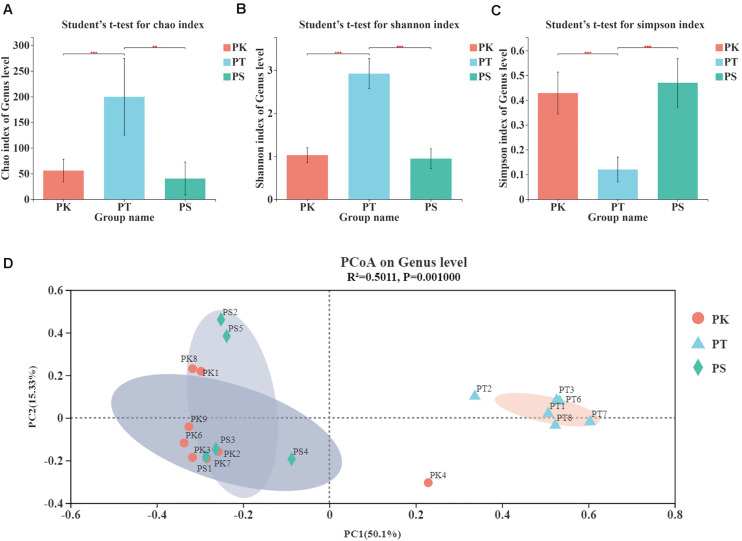
α-diversity and β-diversity of midgut bacteria in *M. saltuarius* feeding on different host trees. **(A)** Significant differences in the Chao index (richness estimator). **(B)** Significant differences in the Shannon index (diversity estimator). **(C)** Significant differences in the Simpson index (diversity estimator) (Student’s *t*-test; * *P* < 0.05, **P < 0.01, ***P < 0.001). **(D)** Principal coordinate analysis based on bray_curtis distances generated from OTU tables. Ovals of different colors represent different groupings (adonis; *P* = 0.001).

The α-diversity of samples at the OTU level was estimated by the Chao, Simpson, and Shannon indices (Figure 2; *P*-values and false discovery rates are shown in Supplementary Table 4). The Chao and Shannon indices of midguts of larvae feeding on *P. koraiensis* and *P. sylvestris* var. *mongolica* (PK and PS) were both significantly lower than those of *P. tabuliformis* (PT; Student’s *t*-test, *P* < 0.01; Figures 2A,B), and the Simpson indices of PK and PS were both significantly greater than that of PT (Student’s *t*-test, *P* < 0.001; Figure 2C). The α-diversity of these groups suggested that species richness and community microorganism diversity in PK and PS were similar, but both were greater in the larvae feeding on *P. tabuliformis*.

The β-diversity of samples at the OTU level illustrated similarities and differences in species composition and community structure. According to the principal coordinate analysis (Figure 2D), microbial communities in all samples were divided into two clear groups (adonis, *R*^2^ = 0.5011, *P* = 0.001). We found that PK and PS groups clustered, and PT was an independent group. These results showed that larvae feeding on *P. koraiensis* and *P. sylvestris* var. *mongolica* shared a similar microbial composition in their midgut. However, larvae feeding on *P. tabuliformis* differed from other samples in microbial composition.

### Comparison of Microbial Community Composition of Different Host Plant Diet at Different Taxonomic Levels

**FIGURE 3:**
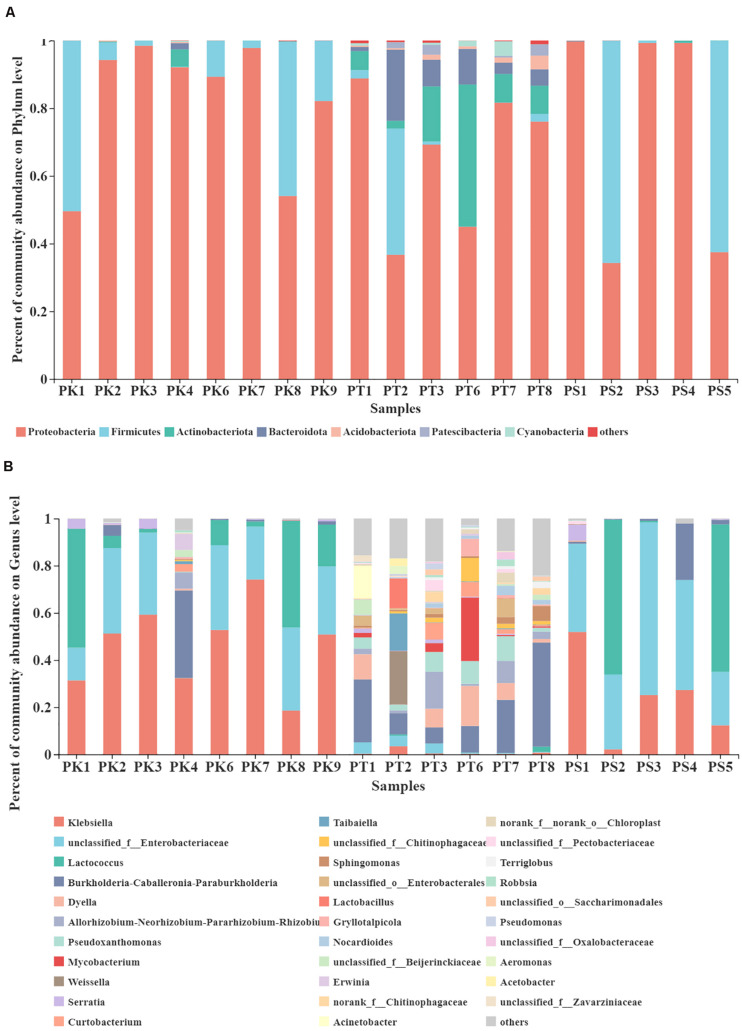
Taxonomic composition of highly abundant bacterial phyla and genera in the gut microbiota of larvae feeding on different host trees. **(A)** Relative abundance of dominant microbial phyla (abundance ≥ 1%). **(B)** Relative abundance of dominant microbial genera (abundance ≥ 2%). The relative percent abundance of bacterial genera is represented by different colors.

A total of 28 phyla, 61 classes, 144 orders, 237 families, and 441 genera were detected in 852 OTUs. The community composition of each sample was analyzed at the phylum and genus level. Bar plots show the percent of community abundance at different taxonomic levels, while species with abundance < 1% at the phylum level and 2% at the genus level are denoted as “others” (Figure 3). The phyla Proteobacteria, Firmicutes, Actinobacteriota, and Bacteroidota were the four most abundant phyla in intestinal samples from larvae feeding on *P. koraiensis*, which were similar to samples from larvae feeding on *P. sylvestris* var. *mongolica*, with only three abundant phyla: Proteobacteria, Firmicutes, and Actinobacteriota. However, at the phylum level, bacteria associated with samples from larvae feeding on *P. tabuliformis* showed greater diversity; the seven most abundant bacterial communities were Proteobacteria, Actinobacteriota, Bacteroidota, Firmicutes, Acidobacteriota, Patescibacteria, and Cyanobacteria (Figure 3A). At the genus level, 32 highly abundant genera were detected in all samples (Figure 3B). The bacterial genera from *M. saltuarius* midguts showed different distribution according to the host tree species. We found that the top four genera with an average abundance of > 1% in *P. koraiensis* feeders were *Klebsiella*, unclassified*_f__Enterobacteriaceae*, *Lactococcus*, and *Burkholderia–Caballeronia–Paraburkholderia*. Similar to *P. koraiensis* feeders, highly abundant genera in intestines of *P. sylvestris* var. *mongolica* feeders were consistent, but with different abundance levels; the relative abundance from high to low was unclassified_f__*Enterobacteriaceae*, *Lactococcus*, *Klebsiella*, and *Burkholderia–Caballeronia–Paraburkholderia*. By contrast, we identified 13 genera with an average abundance of > 2% in larvae feeding on *P. tabuliformis*. These 13 genera were *Burkholderia–Caballeronia–Paraburkholderia*, *Dyella*, *Pseudoxanthomonas*, *Mycobacterium*, *Allorhizobium–Neorhizobium–Pararhizobium–Rhizobium*, *Weissella*, unclassified_f_*Chitinophagaceae*, *Taibaiella*, *Curtobacterium*, unclassified_o_*Enterobacterales*, *Sphingomonas*, *Lactobacillus*, and unclassified_f__*Enterobacteriaceae*. *P. tabuliformis* feeders showed the most remarkable intrasample microbiota diversity.

**FIGURE 4:**
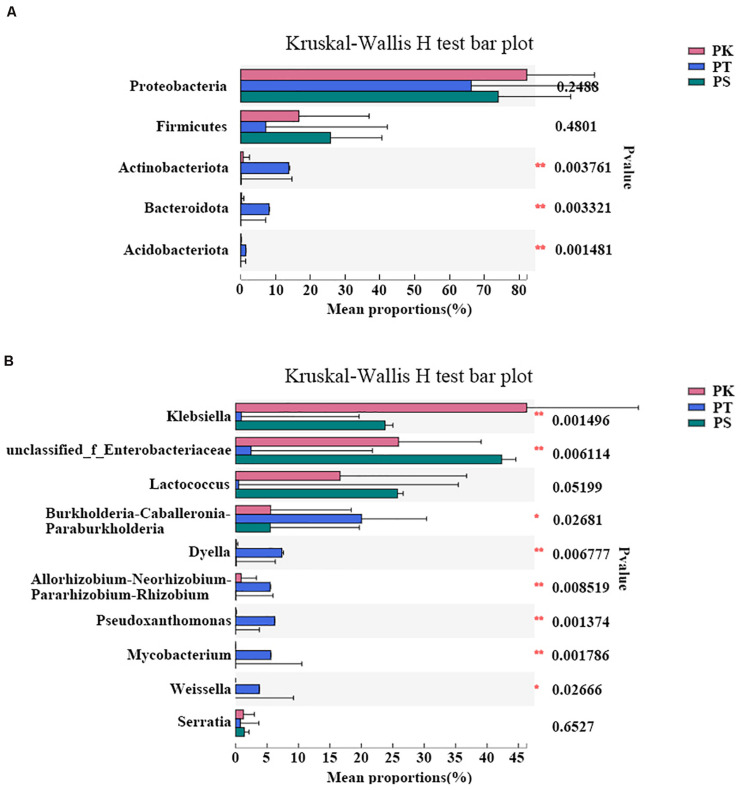
Differences in intestinal bacteria of *M. saltuarius* feeding on different host tree species at the phylum and genus level. **(A)** Differences in abundance of dominant bacterial phyla (the top five are shown). **(B)** Differences in abundance of dominant bacterial genera (the top ten are shown) (Kruskal–Wallis *H*-test; *P < 0.05, **P < 0.01, *** *P* < 0.001).

In order to further understand whether the host tree species have a significant impact on the composition of gut bacteria of larvae, ANOSIM analysis was carried out in all collected samples (Supplementary Table 5): At the phylum level, the composition of gut bacteria showed significant differences (*P* < 0.05) according to different diets, while most significant at the genus level (*P* = 0.001). To identify different abundances at phylum and genus levels, the significance levels of differences were tested in all samples (Figure 4). All sample groups had significant differences in abundance in the top five phyla with the greatest abundance, except for Proteobacteria and Firmicutes (Kruskal–Wallis *H*-test, *P* < 0.05; Figure 4A). Furthermore, of the top ten genera with the greatest abundance, all genera showed a significantly different abundance in each sample group, except for *Lactococcus* and *Serratia* (Kruskal–Wallis *H*-test, *P* < 0.05; Figure 4B). *Klebsiella* (*P* = 0.002) was the most dominant bacterial genus in *P. koraiensis* feeders, while levels of unclassified_f__*Enterobacteriaceae* (*P* = 0.006) were greatest in *P. sylvestris* var. *mongolica* feeders. Furthermore, six genera, *Burkholderia*–*Caballeronia*–*Paraburkholderia* (*P* = 0.027), *Dyella* (*P* = 0.007), *Allorhizobium*–*Neorhizobium*–*Pararhizobium*–*Rhizobium* (*P* = 0.009), *Pseudoxanthomonas* (*P* = 0.001), *Mycobacterium* (*P* = 0.002), and *Weissella* (*P* = 0.027), showed greater abundance in *P. tabuliformis* feeders than in samples from *P. koraiensis* and *P. sylvestris* var. *mongolica* feeders.

### Functional Predictions for *M. saltuarius* Gut-Associated Bacterial Communities

**FIGURE 5:**
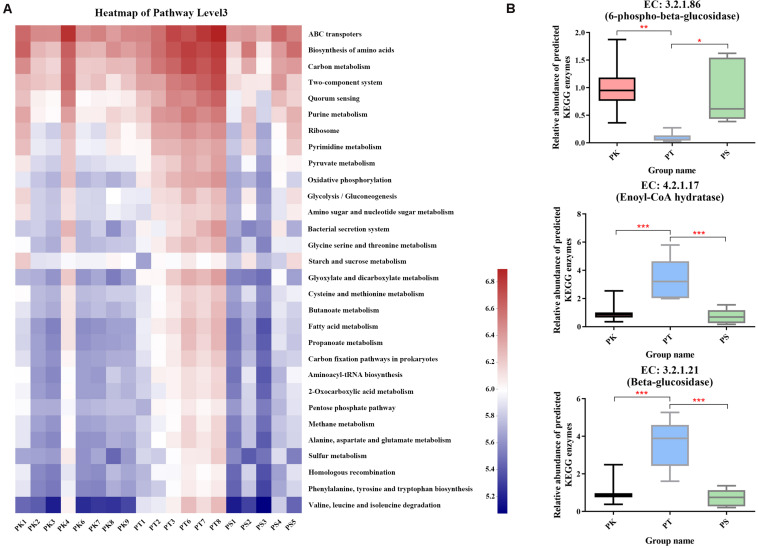
Comparison of predicted KEGG ortholog group counts at level 3 and enzyme levels in different host tree species. **(A)** Heat map of the most abundant 30 categories at level 3; each column corresponds to an *M. saltuarius* sample, and each row corresponds to a specific category. **(B)** Relative abundance of three highly abundant predicted KEGG enzymes involved in cellulose or limonene and pinene degradation in the guts of larvae feeding on different host tree species (one-way ANOVA; *P < 0.05, **P < 0.01, ***P < 0.001).

**FIGURE 6:**
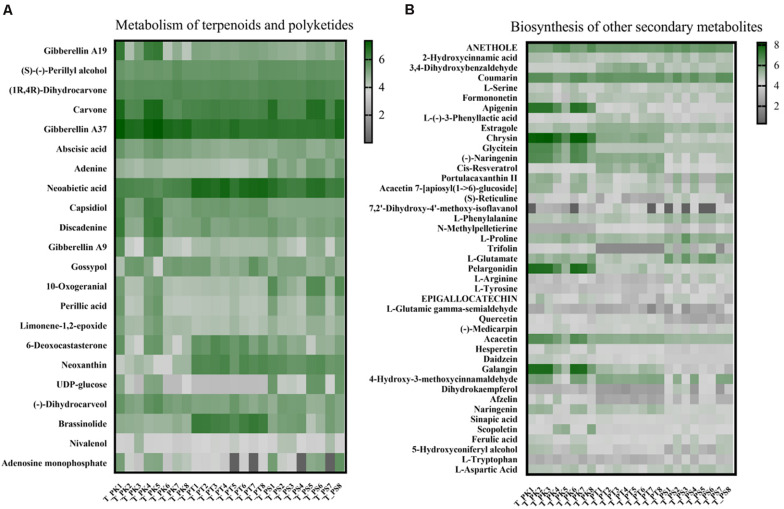
Comparison of relative metabolite abundance in wood tissue from different host tree species. **(A)** Heat map of metabolites annotated in the pathway “metabolism of terpenoids and polyketides.” **(B)** Heat map of metabolites annotated in the pathway “biosynthesis of other secondary metabolites.” Each column corresponds to a host tree wood tissue sample, and each row corresponds to a specific metabolite. T_PK, xylem tissue from *P. koraiensis*; T_PT, xylem tissue from *P. tabuliformis*; T_PS, xylem tissue from *P. sylvestris* var. *mongolica.* All of these abbreviations apply to the following figures.

In KEGG functional annotations, 379 level 3 KEGG pathways were predicted for all samples (Supplementary Table 6). Different patterns in the frequencies of the 30 functions with the greatest abundance in host tree species were identified (Figure 5A). *P. koraiensis* and *P. sylvestris* var. *mongolica* feeder samples were similar, while *P. tabuliformis* feeders exhibiting a greater frequency of metabolic categories were associated with energy metabolism (carbon metabolism and fixation pathways in prokaryotes); translation (aminoacyl-tRNA biosynthesis); metabolism of carbohydrates (amino sugar and nucleotide sugar metabolism); and amino acids (glycine, serine, cysteine, methionine, etc.). In addition, several key enzymes involved in the degradation of cellulose or participating in the degradation of limonene and pinene were also annotated; among the three key highly abundant enzymes (the abundance level was in the top 100) predicted in all samples, the abundances of enoyl-CoA hydratase (EC 4.2.1.17) and β-glucosidase (EC 3.2.1.21) in PK and PS samples were significantly lower than those in PT samples (Figure 5B). By contrast, the abundance of 6-phospho-β-glucosidase (EC 3.2.1.86) in PT samples was significantly lower than that in PK and PS samples (Figure 6B). Some other key enzymes, including cellulose or endoglucanase (EC 3.2.1.4), cellulose 1,4-β-cellobiosidase (EC 3.2.1.91), and cellobiose phosphorylase (EC 2.4.1.20), which are involved in the degradation of cellulose; and α-pinene dehydrogenase or monooxygenase (EC 1.14.-.-), limonene 1,2-monooxygenase (EC 1.14.13.107), and carveol dehydrogenase (EC 1.1.1.243), which participate in the degradation of limonene and pinene, were also annotated.

### Comparisons of Different Metabolite Patterns in Wood Tissue and the Larval Gut

A total of 1,335 and 1610 metabolites were identified, respectively in all xylem tissue samples and larval gut samples; among them 360 and 472 metabolites have been annotated in the KEGG database (xylem tissue samples: Supplementary Table 7; larval gut samples Supplementary Table 8); 22 substances were annotated as participating in terpenoid and polyketide metabolism; 43 metabolites played a role in biosynthesis of other secondary metabolites. To visualize differences in the wood tissue metabolome associated with different host tree species, we assessed the relative abundance of metabolites involved in the above pathways with a heat map (Figure 6). Among the 22 substances annotated as involved in terpenoid and polyketide metabolism, the relative abundances of (1*R*, 4*R*)-dihydrocarvone, adenine, capsidiol, and 10-oxogeranial in *P. sylvestris* var. *mongolica* were significantly different from those in the other two species; 6-deoxocastasterone and brassinolide were significantly enriched in xylem tissue samples from *P. tabuliformis*; and the relative abundances of only gibberellin A37 and neoxanthin were significantly different in *P. koraiensis* compared with those in the other two tree species (Supplementary Table 9). The significance test in the abundance of compounds in the biosynthesis pathway of other secondary metabolites within three host-tree species between groups was also tested (Supplementary Table 10).

**FIGURE 7:**
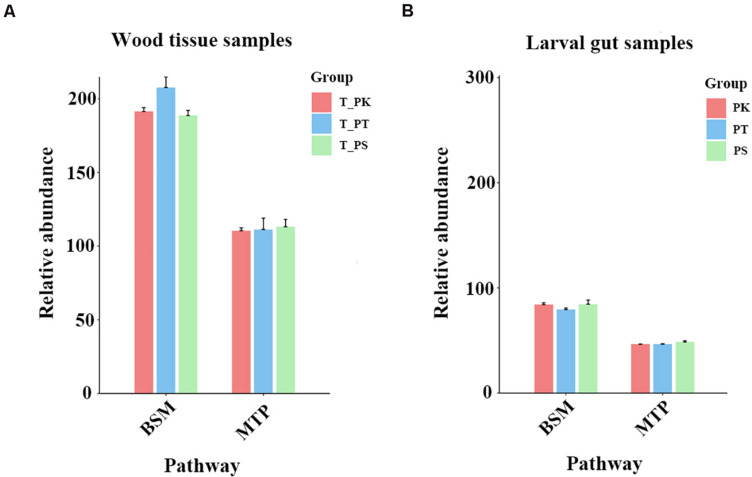
Comparison of relative abundances of compounds annotated in three KEGG pathways. **(A)** Wood tissue samples. **(B)** Larval gut samples. BSM, biosynthesis of other secondary metabolites; MTP, metabolism of terpenoids and polyketides. Relative abundances were calculated by the sum normalization method.

To a deeper understanding of overall differences in putative plant defense substances in xylem tissue samples from different host trees and their possible varieties in different diets in larval guts, xylem samples from different host tree species and intestinal samples from larvae feeding on different host tree species were assessed separately. Two related KEGG pathways (biosynthesis of other secondary metabolites and metabolism of terpenoids and polyketides) were selected, and the relative abundances of all compounds annotated in these pathways were compared individually (Figure 7). We did not find a significant difference in the total relative abundance of compounds annotated in the pathway “metabolism of terpenoids and polyketides” (Figure 7). However, the relative abundance of substances in *P. tabuliformis* xylem samples in the pathway “biosynthesis of other secondary metabolites” was apparently greater than those in *P. koraiensis* and *P. sylvestris* var. *mongolica* samples (Figure 7A). Compared with the intestinal samples of larvae feeding on *P. koraiensis* and *P. sylvestris* var. *mongolica*, samples from *P. tabuliformis* feeders had a slightly lower relative abundance of substances in the pathway “biosynthesis of other secondary metabolites” (Figure 7B).

### Correlation Analysis of Bacterial Genera and Gut Metabolites

**FIGURE 8:**
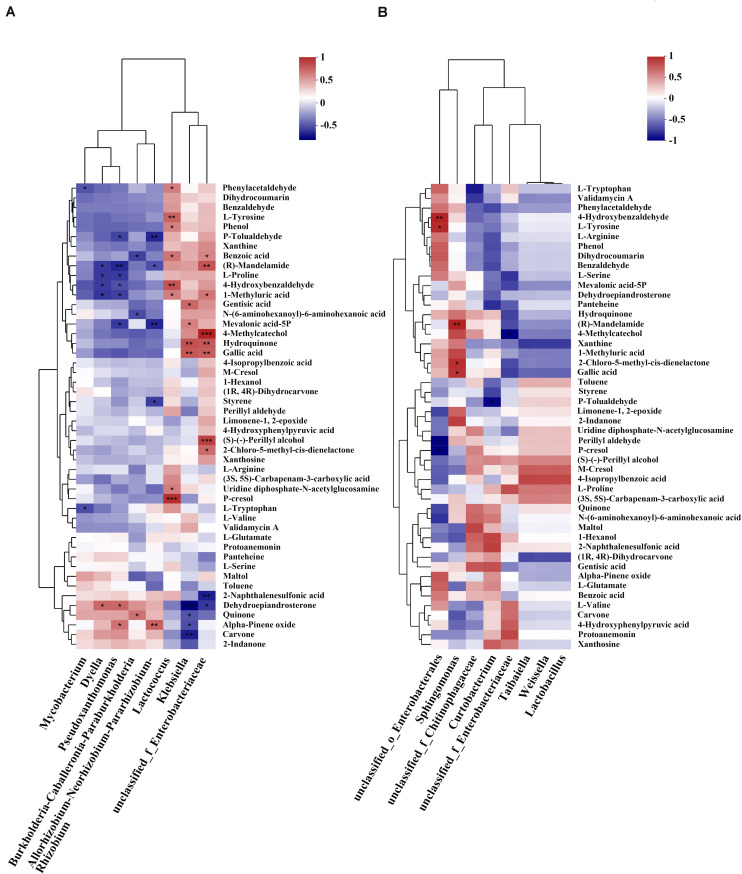
Correlation analysis between genera abundant in the gut and gut metabolite contents annotated as putative plant defense substances. **(A)** Correlations between the top five abundant genera in larvae feeding on all host tree species and compounds annotated as related to plant defense substances. **(B)** Correlations between abundant genera in *P. tabuliformis* feeders (abundance ≥ 2%, except for the top five) and putative plant defense metabolites. Each row in the figure represents a metabolite, each column represents a genus, and each lattice represents a Pearson correlation coefficient between a component and a metabolite. Red represents positive correlations, while blue represents negative correlations (*P < 0.05, **P < 0.01, ***P < 0.001).

As shown in Figure 8A, among the bacterial communities with a relatively high abundance in larval guts from *P. koraiensis* and *P. sylvestris* var. *mongolica* feeders, the genus *Lactococcus* was positively associated with phenylacetaldehyde, L-tyrosine, phenol, benzoic acid, 4-hydroxybenzaldehyde, 1-methyluric acid, uridine diphosphate-*N*-acetylglucosamine, and *p*-cresol. The genus *Klebsiella* was positively associated with gentisic acid, mevalonic acid-5P, hydroquinone, and gallic acid, but negatively associated with dehydroepiandrosterone, quinone, α-pinene oxide, and carvone. The genus unclassified_f__*Enterobacteriaceae* was positively correlated with many metabolites annotated as putative plant defense substances and negatively correlated with two metabolites. The genus *Burkholderia*–*Caballeronia*–*Paraburkholderia*, which was highly abundant in intestinal samples from larvae feeding on all three host tree species, was negatively associated with benzoic acid and *N*-(6-aminohexanoyl)-6-aminohexanoic acid, and positively associated with quinone. Within abundant genera from samples from *P. tabuliformis* feeders, the genus *Mycobacterium* was only negatively associated with phenylacetaldehyde and L-tryptophan, while the genus *Allorhizobium*–*Neorhizobium*–*Pararhizobium*–*Rhizobium* was negatively associated with *p*-tolualdehyde, (*R*)-mandelamide, mevalonic acid-5P, and styrene, but positively associated with α-pinene oxide. The genus *Dyella* (an abundant genus in samples from all host tree feeders) and the genus *Pseudoxanthomonas* (a dominant genus in samples from *P. tabuliformis* feeders) harbored similar correlations with (*R*)-mandelamide, L-proline, 4-hydroxybenzaldehyde, and 1-methyluric acid (negative correlations), and dehydroepiandrosterone (positive correlation). As shown in Figure 8B, among the bacterial communities abundant in samples from *P. tabuliformis* feeders, the genera *Taibaiella*, *Weissella*, and *Lactobacillus* had a strikingly similar correlation with almost all plant defense-related substances. The genus *Sphingomonas* was positively correlated with many plant defense substances and significantly correlated with (*R*)-mandelamid, 2-chloro-5-methyl-*cis*-dienelactone, and gallic acid. Moreover, the genus unclassified_o__*Enterobacterales* was positively associated with 4-hydroxybenzaldehyde and L-tyrosine, which are involved in the biosynthesis of other secondary metabolites (Figure 8B), while the genera unclassified_f__*Chitinophagacae*, *Curtobacterium*, and unclassified_f__*Enterobacteriaceae* had few significantly correlated metabolites.

## Discussion

In this study, we described and compared, for the first time, the gut microbiota in *M. saltuarius* feeding on different host trees based on quantitative PCR and 16S rDNA gene amplicon sequencing. The intestinal larval metabolism of different host tree diets and xylem metabolism of different host tree species were added to reveal differences in metabolites in xylems of different tree species and intestinal contents from different diets. A joint analysis of the dominant bacterial genera in larval intestines and gut metabolites of intestinal contents was conducted to determine the correlation between gut microbiota and potential plant defense metabolites.

### Host Tree Species Influencing the Gut Bacteria in *M. saltuarius* Larva

Microbiome variation in the insect gut is influenced foremostly by diet composition (Mason, 2020); Larvae with similar diet may have similar gut bacteria, but show great differences in different hosts. In this study, the average number of bacterial 16S rRNA genes was higher than 1e + 08 copies/g in all three groups, which indicate the midgut associate bacteria may have enough ability to benefit host metabolize. However, there was no significant difference in qPCR results between groups, which means that host plants may not be an important factor affecting the total amount of insect’s midgut bacteria. On the contrary, bacterial communities in *M. saltuarius* gut varied significantly between groups on both phylum and genus levels, indicating that the host species could influence the microbial community, which was in agreement with previous findings in other insects (Colman et al., 2012; Dematheis et al., 2012; Chu et al., 2013; Jones et al., 2013; Yun et al., 2014; Chung et al., 2017; Blankenchip et al., 2018; Santos-Garcia et al., 2020; Wang et al., 2020). Under the bray curtis, the distance between PK and PS samples seemed closer, indicating that there might be more similar species shared between these groups; while compared to PT group, the bacterial communities clustered further apart, thus indicating that larval populations developing on *P. tabuliformis* harbored greater microbial diversity than did populations developing on the other two host tree species, and presented a large fraction of *P. tabuliformis*-specific species.

Bacterial phyla in *M. saltuarius* larval gut were dominated by Proteobacteria and Firmicutes. Represented by *Monochamus* and *Anoplophora* species, these typical taxa were repeatedly reported as dominant phyla in the guts of many Cerambycidae species (Schloss et al., 2006; Rizzi et al., 2013; Vicente et al., 2013; Hu et al., 2017; Kim et al., 2017; Chen et al., 2020), and are even the predominant phyla in the guts of insects (Yun et al., 2014). The high abundance levels of these phyla may be due to active recruitment by insects, or to taxa of these phyla being more likely than other bacterial communities to invade and colonize insect hosts (Jones et al., 2013). On the contrary, high abundant genera in the larval guts were quiet diverse according to different host plants: in the guts of *P. koraiensis* and *P. sylvestris* var. *mongolica* feeders, *Klebsiella*, unclassified*_f__Enterobacteriaceae*, *Lactococcus*, and *Burkholderia–Caballeronia–Paraburkholderia* were most dominant, these typical taxa are often thought ro be associated with xylophagous insects (Reid et al., 2011; Morales-Jiménez et al., 2012; Rizzi et al., 2013; Berasategui et al., 2017; Chen et al., 2020), as they can associate with their host by supplementing specifically required forms of nitrogen and other nutrients thus ensure a normal development (Behar et al., 2005). In addition, *Burkholderia* species are terpene-degrading species [e.g., *Burkholderia xenovorans* degrades diterpene resin acids and can utilize diterpenes as a sole carbon source (Smith et al., 2007)], and some symbionts in this genus also have fenitrothion-degrading capabilities (Kikuchi et al., 2012). The existance of these bacteria might be benefical for larvae to face a variety of plant chemical defense substances. While in samples from *P. tabuliformis* feeders there were 13 bacterial genera with a relatively high abundance, among these *P. tabuliformis* feeders specific symbionts, they have not only been reported to have the ability of nitrogen fixation and cellulose metabolism, but also the ability to metabolize a variety of complex compounds (Nayak et al., 2011; Ahn et al., 2012; Engel and Moran, 2013; Choi et al., 2015), which might be benefificial for *P. tabuliformis* feeders to adapt better to the environment.

Moreover, some dominant bacterial genera in the gut of *M. saltuarius* larvae were also reported in soil and pine tissues (e.g., *Burkholderia*, *Dyella*, and *Mycobacterium*) (Guo et al., 2020), and there are also examples in previous studies showing that insects can obtain beneficial microbiota from the environment to better adapt to the host (e.g., *Bemisia tabaci* improves its fitness on unsuitable hosts by changing the composition and abundance of its gut microbiota) (Santos-Garcia et al., 2020). These findings raise the possibility that some environmental bacteria can establish a beneficial symbiotic relationship with longhorn beetles, and some of them can become consistent gut residents. It is noteworthy that the genus *Mycobacterium* shown a relatively high abundance in *P. tabuliformis* feeders from which a genus believed to play an important role in the adaptation of insects to less suitable hosts (Santos-Garcia et al., 2020). *Mycobacterium* species have high levels of environmental adaptability; by producing biofilms, they can adhere to different surfaces, and also overcome unfavorable abiotic and biotic environments (Esteban and García-Coca, 2017). More than that, some *Mycobacterium* species have extremely powerful bioremediation capabilities and can degrade many complex compounds, e.g., alkanes and isoprenoids (Berekaa and Steinbüchel, 2000).

### Different Host Tree Metabolites and the Possible Role of Larval Gut Bacteria in Adapting to Unfavorable Hosts

Differences of gut bacterial genera in different host tree feeders at the taxonomic level reflect aspects of the host tree species, which are all natural hosts of the beetles, and we infer that different intestinal bacterial compositions help longhorn beetle larvae to adapt diverse host metabolite characteristics. Although large differences in composition and abundance of the dominant bacterial genera existed between different host species feeders, the dominant gut bacterial genera from different host tree feeders were reported as similar metabolic functions (Morales-Jiménez et al., 2012; Rizzi et al., 2013; Berasategui et al., 2017), diverse genera between groups may as counterparts to compensate a same type of nutrients supplement or metabolism activities, while there might be differences in specific capabilities.

Our metabolomic data revealed that the concentrations of many compounds in the xylems of three host tree species are significantly different, thus means a diverse environment of metabolites faced by larvae in different host tree species, which may amplify the difference of gut bacteria between *P. tabulaeformis* feeder’s and the others. As a less suitable host, *P. tabuliformis* xylems showed a higher concentration of “biosynthesis of other secondary metabolites” involved substances. Whereas, similar compounds shown a relatively low abundance in the gut of *P. tabuliformis* feeders, which indicates that *P. tabuliformis* feeding larvae harbor a greater ability to metabolize plant secondary metabolites. According to the results of the joint correlation analysis and functional predictions, dominant bacterial genus in *P. tabuliformis* feeders shown more negatively correlations with a large number of substances, and were predicted as stronger metabolic capacity, thus we infer that dominant gut bacteria in *P. tabuliformis* feeders harbors stronger metabolic capacity of potentially toxic compounds. Joint correlation analysis between gut bacterial genera with abundance ≥ 2% (except for the top five genera) in *P. tabuliformis* feeders and related metabolites showed that these bacteria were not more negatively correlated with metabolites that related to plant defense, and only three bacterial genera (*Curtobacterium*, unclassified_f__*Enterobacteriaceae*, and unclassified_o__*Enterobacterales*) were significantly negatively correlated with a very limited number of metabolites. Based on this, we inferred that these bacteria may provide nutritional supplementation to their insect hosts at a limited level. However, even the larvae on *P. tabuliformis* with a diverse bacteria which may metabolize more potentially toxic compounds and provide limited nutritional supplement, compared to other tree species, *P. tabuliformis* is not a host that larvae preferentially choose to feed on or lay eggs on in nature (Hwang et al., 2008; Pan et al., 2020), thus we hypothesized that the dominant gut bacteria of *P. tabuliformis* feeders only play a certain metabolic or compensatory role, but cannot eliminate all negative effects of the host.

As the pathogenic nematode of a devastating disease, the life cycle of *B. xylophilus* and the interaction between nematode and insect vectors have received extensive attention for a long time (Kikuchi et al., 2011; Li et al., 2021). Nematode larvae can secrete ascarosides which promote the pupation of the last instar *Monochamus* larvae, while adult beetles secrete the same substance to attract the dispersal fourth-stage nematode LIV larvae and transmit them to new trees (Zhao et al., 2016). In this study, to avoid the effect of overwintering stage on the gut bacteria of the larvae, only 3rd and 4th instar larvae were collected in Aug and Sep, which the stage are generally thought not to associate with nematodes. However, the presence of nematodes is likely to affect the microbial symbionts of pupae and adults of insects. At present, few studies have explored the effect of nematode on insect microbial symbioses, and the role of these microbial symbionts in the infection cycle of pine wilt diease is still unclear. Therefore, our follow-up research will focus on revealing the dynamics of symbiotic microorganisms in insects after nematode infection, as well as the biological function of important microbial symbioses.

## Conclusion

Although we did not conduct an *in vitro* culture experiment on longicorn larval intestinal bacteria, the combination of our existing experiments suggests that: compared with the other two tree species, *P. tabuliformis* contains higher concentrations of and more diverse secondary metabolites, which make it a less suitable host tree species for *M. saltuarius* larvae. Furthermore, in the intestines of larvae that feed on *P. tabuliformis*, a diverse gut microbiota contributes to a greater ability to metabolize secondary plant metabolites, which benefits the host larvae (Hammer and Bowers, 2015; Shikano et al., 2017). These diverse bacteria may be acquired from host trees or the environment, and plant metabolites may also shape the gut microbiota. However, despite the existence of the insect-beneficial bacteria mentioned above, the negative effect of *P. tabuliformis* as an unsuitable host on larval growth and development cannot be completely eliminated. Further experiments and analyses are required to determine the exact origin of the diverse gut microbiota and the contribution of each component to host tree species adaptations.

## Data Availability Statement

The datasets presented in this study can be found in online repositories. The names of the repository/repositories and accession number(s) can be found below: NCBI, Submission ID: SUB9169080, BioProject ID: PRJNA705565.

## Author Contributions

S-XG, S-XZ, and L-LR designed the study. S-XG performed the experiments, analyzed the data, and wrote the manuscript. F-MS, J-HP, and Z-HH provided help with the experiments. S-XG and L-LR discussed the results and edited the manuscript. All authors read and approved the final manuscript.

## Conflict of Interest

The authors declare that the research was conducted in the absence of any commercial or financial relationships that could be construed as a potential conflict of interest.

## Footnotes

1 https://cloud.majorbio.com

2 http://www.hmdb.ca/

3 https://metlin.scripps.edu/

4 http://bioconductor.org/packages/release/bioc/html/ropls.html

5 https://cloud.majorbio.com

6 http://www.genome.jp/kegg/

## Supplementary Materials

Supplementary Figure 1 Rarefaction curves of bacterial communities in the intestine samples of *Monochamus saltuarius* fed on different host tree species. Sobs: number of species observed in the sample; PK: mid-guts of *P. koraiensis* fed larvae; PT: mid-guts of *P. tabuliformis* fed larvae; PS: mid-guts of *P. sylvestris* fed larvae.

Supplementary Figure 2 Mean qPCR values [copies bacterial 16S rRNA gene (rRNA/g)] across all groups of different host-tree species feeding larvae.

Supplementary Table 1 Summary statistics for the Illumina Miseq runs for all samples.

Supplementary Table 2 Summary statistics for the qPCR runs for all samples.

Supplementary Table 3 Test data of Differences between groups in Mean qPCR values (Tukey’s multiple comparisons test).

Supplementary Table 4 Test data of Differences between groups in α-diversity.

Supplementary Table 5 Test data of ANOSIM analysis between groups for all samples.

Supplementary Table 6 PICRUST2 Functional annotations of KEGG pathway level 3 in all samples.

Supplementary Table 7 Metabolites identified in wood tissue samples and annotated information.

Supplementary Table 8 Metabolites identified in larval gut samples and annotated information.

Supplementary Table 9 Significance test of relative abundance in substances annotated in metabolism of terpenoids and polyketides (Kruskal–Wallis *H*-test).

Supplementary Table 10 Significance test of relative abundance in substances annotated in biosynthesis of other secondary metabolites (Kruskal–Wallis *H*-test).
