# Supplementary figures and images for: Gut Bacteria Associated With Monochamus saltuarius (Coleoptera: Cerambycidae) and Their Possible Roles in Host Plant Adaptations
Source: Front Microbiol. 2021 Jun 21;12:687211. doi: 10.3389/fmicb.2021.687211 (PMC8256174; doi:10.3389/fmicb.2021.687211)

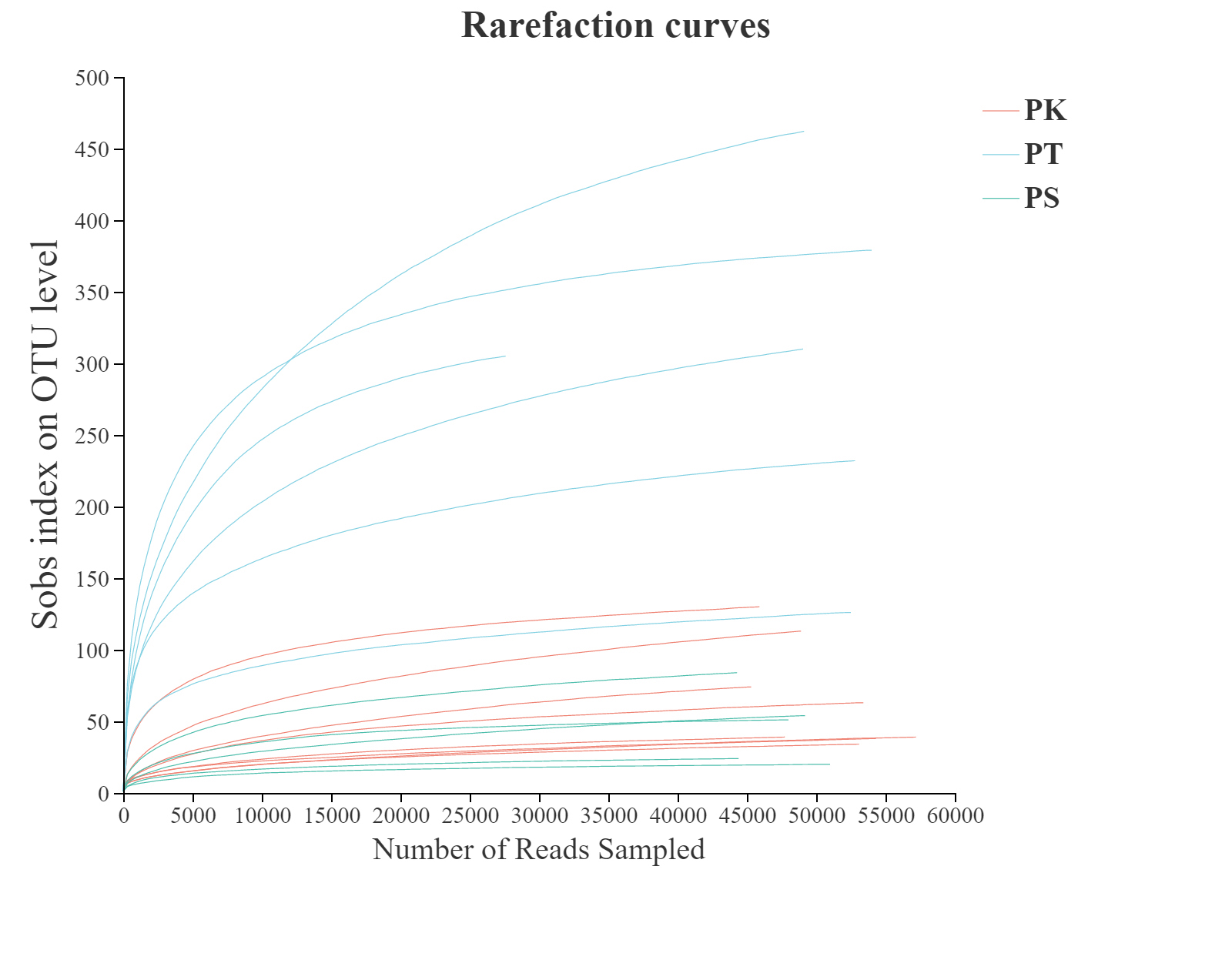

Supplement: Supplementary Figure 1 — Rarefaction curves of bacterial communities in the intestine samples of Monochamus saltuarius fed on different host tree species. Sobs: number of species observed in the sample; PK: mid-guts of P. koraiensis fed larvae; PT: mid-guts of P. tabuliformis fed larvae; PS: mid-guts of P. sylvestris fed larvae. [file Image_1.JPEG]

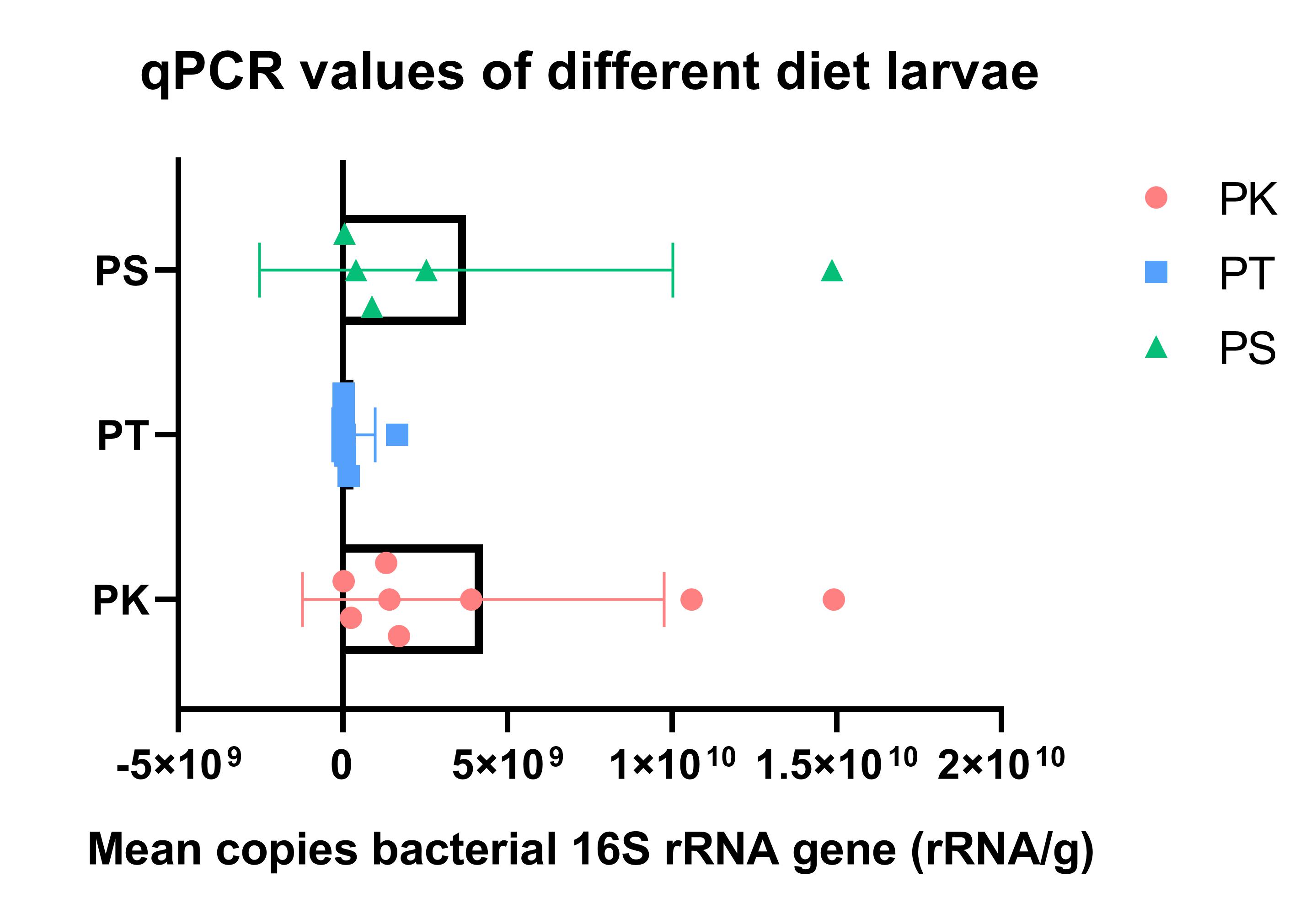

Supplement: Supplementary Figure 2 — Mean qPCR values [copies bacterial 16S rRNA gene (rRNA/g)] across all groups of different host-tree species feeding larvae. [file Image_2.JPEG]
